# Mechanism of community quitters’ psychological traits on their smoking cessation effects: Based on a study of community intervention

**DOI:** 10.18332/tid/162000

**Published:** 2023-05-26

**Authors:** Xinran Huo, Xingming Li, Mingyu Gu, Tingting Qin, Kun Qiao, Xinyuan Bai, Yao Wang, Yutong Yang

**Affiliations:** 1 School of Public Health, Capital Medical University, Beijing, China

**Keywords:** smoking cessation, psychological intervention, psychological traits, influence factor, smoking cessation effects

## Abstract

**INTRODUCTION:**

We study the relationship between psychological traits of smokers and their smoking cessation effects, and provide more scientific evidence for smoking cessation intervention.

**METHODS:**

The study was conducted as a nested case-control study. Smokers who participated in the community smoking cessation intervention projects in Beijing in 2018–2020, were selected as the research participants and divided into two groups: a successful smoking cessation and unsuccessful smoking cessation group, according to their smoking cessation effects at 6 months. Psychological traits of quitters including smoking abstinence self-efficacy, willingness to quit smoking, and trait coping style, were compared between the two groups, and a structural equation model was established for confirmatory factor analysis to analyze their mechanisms.

**RESULTS:**

There were differences in smoking cessation results between the successful smoking cessation group and the unsuccessful smoking cessation group in terms of smoking abstinence self-efficacy and willingness to quit smoking. Willingness to quit smoking (OR=1.06; 95% CI: 1.008–1.118) is a risk factor, while smoking abstinence self-efficacy in habit/addiction situations (OR=0.77; 95% CI: 0.657–0.912) is a protective factor. The results of the structural equation model showed that smoking abstinence self-efficacy (β=0.199, p=0.002) and trait coping style (β= -0.166, p=0.042) could influence smoking cessation effects. The structural equation model was well fitted, which showed that smoking abstinence self-efficacy (β=0.199, p=0.002) and trait coping style (β= -0.166, p=0.042) might have influenced the effect of smoking cessation among smokers.

**CONCLUSIONS:**

Willingness to quit smoking has a positive impact on the smoking cessation effect, while smoking abstinence self-efficacy in habit/addiction situations and negative trait coping style have a negative impact. Smoking abstinence self-efficacy and trait coping styles can significantly affect smoking cessation outcomes.

## INTRODUCTION

The results from the 2018 National Adult Tobacco Epidemic Survey show that the number of smokers in China exceeds 300 million, the smoking rate among people aged ≥15 years was 26.6%, the rate of adult men smokers was 50.5%^1^, and about 8 million people lose their lives to tobacco use each year, of which about 7 million die from diseases caused by smoking and about 1.2 million die from diseases caused by secondhand smoke exposure^2^. WHO also included tobacco dependence as a disease in the International Classification of Diseases (ICD-10, F17.2), confirming that tobacco is currently the greatest threat to human health^3^.

Studies have shown that social, psychological, and physiological factors all have some degree of influence on smoking cessation effects, with psychological traits being the key to quitting smoking^4^. Psychological traits are unique characteristics of each individual, namely the psychological profile, and they are expressed through relatively stable behaviors that are specific to the individual. As for smoking cessation, the success rate of smoking cessation is closely related to smokers’ psychological traits, such as willingness to quit and smoking abstinence self-efficacy^5^, which also provide important conditions for smoking behavior change.

Studies have shown that people are prone to smoke when they have negative emotions or under massive psychological pressure, which is significantly associated with a low success rate of quitting^6^. However, when people are under pressure or have negative emotions, negative coping strategies such as denial and avoidance can lead them to smoke^7^. Therefore, we hypothesized that individuals who are often in passive avoidance or a pessimistic state of mind would be less likely to achieve successful cessation.

Self-efficacy is significantly associated with cessation results^8^, and the increase in smoking abstinence self-efficacy can improve the likelihood of quitting and reducing relapse^9^. So, we need to test whether there was a relationship between self-efficacy for smoking cessation and quitting. Willingness to quit also plays an important role in smoking cessation, and studies have found that it is the basis for successful smoking cessation, which greatly influences the effects of quit attempts^10^. Therefore, the hypothesis that individuals with a stronger desire to quit smoking would be more likely to be successful in quitting, needs to be verified.

Supported by social psychological theories, the effectiveness of Bandura’s self-efficacy theory^11^ and Bern’s self-perception theory^12^ has been demonstrated in clinical and community interventions. Hanqiao et al.^13^ analyzed the influence of individual psychological trait factors on nicotine dependence and found that individual smoking abstinence self-efficacy and trait coping style have an impact on the degree of nicotine dependence of quitters, but there is a lack of research on the mechanisms and pathways of influence of individual psychological trait factors of smokers on their cessation effects.

Therefore, it is helpful to understand the psychological state and psychological traits of quitters in the process of smoking cessation, to analyze the mechanism of the influence of different psychological traits on the cessation effect of quitters, to study and evince the relationship between psychological traits of quitters and their smoking cessation effects, evince associations and point out their directionality.

## METHODS

### Study environment

The baseline was conducted from December 2018 to June 2020. With convenience sampling, 19 communities were selected from 6 districts in Beijing. Each community health service center hosted community lectures, smoking cessation mobilization, and quitter recruitment activities in their area. In all, 683 smokers were screened for inclusion and exclusion criteria to establish a community intervention follow-up cohort, with a comprehensive tobacco dependence management model for the test group and a 2A+R brief smoking cessation intervention for the control group, referring to published literature for the specific intervention protocol^14^.

**Figure 1:**
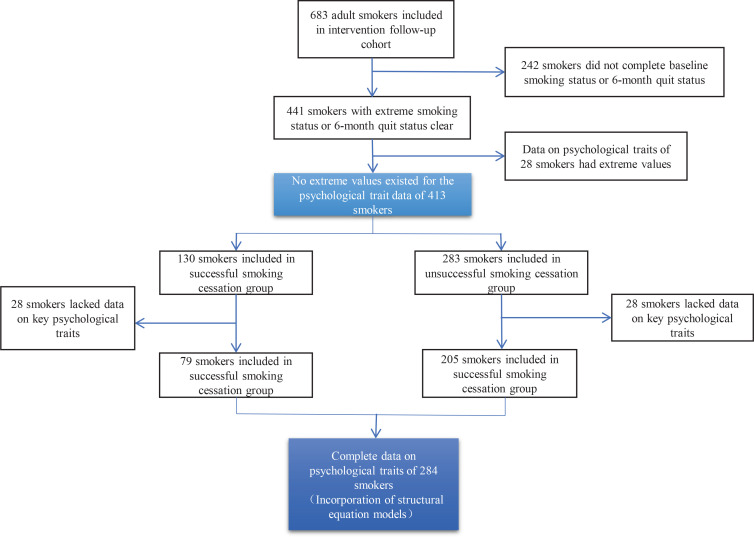
Flow chart of sample size change of study subjects

The study was conducted as a nested case-control study, with a sample forming the control group at a ratio close to 1:2 between the case and control group. The smokers who did not specify whether they had successfully quit were excluded from the intervention follow-up cohort, which included 683 smokers, and the outliers were cleaned out from the survey data, resulting in a total of 413 smokers included in this study (Figure 1). In this study, the 7-day point prevalence abstinence was used as the outcome indicator, and those who had quit smoking after 6 months of enrollment were included in the successful group of 130 residents, while 283 residents who had not quit smoking after assessment were included in the unsuccessful group.

### Study content

According to the literature, various psychological theories, including social, cognitive, and behavioral, could influence smoking behavior and cessation effects^15-19^. The questionnaire consisted of six sections: smoker’s smoking status, cessation behavior, assessment of tobacco harm knowledge, assessment of supportive environment for tobacco control, willingness to quit interventions, psychological assessment, and basic demographic information. The study used a six-part questionnaire: Self-administered Sociodemographic Questionnaire, Smoking Abstinence Self-Efficacy (SASE), Trait Coping Style Questionnaire (TCSQ), Self-administered Willingness to Quit Smoking Scale, and Cessation Effects Indicators.

*Self-administered Sociodemographic Questionnaire*

It included information on age, gender, marital status, education level, average monthly income, hukou household-registration, type of work, awareness of smoking hazards, willingness to quit, etc.

*Smoking Abstinence Self-Efficacy (SASE)*

The scale consists of three contextual dimensions with nine items to measure the smoking abstinence self-efficacy of the research participants. The scale was scored on a 5-point Likert scale: 1 = ‘extremely want to smoke’, 2 = ‘very want to smoke’, 3 = ‘somewhat want to smoke’, 4 = ‘not really want to smoke’, and 5 = ‘not want to smoke at all’. The higher the score, the higher the smoking abstinence self-efficacy. Cronbach’s α for this scale was 0.884, indicating good internal consistency (Table 1).

**Table 1:** Psychological trait variables and assignments^20-23^

*Observed variables*	*Definition*	*Assignment*	*Scale*	*Cronbach’s α*
Positive smoking abstinence self-efficacy	The extent to which the smoker wants to smoke when being in an active or socialized situation	Want to smoke: 1=extremely 2=very 3=somewhat 4=not really 5=not at all	Smoking abstinence self-efficacy	0.884^22,23^
Negative smoking abstinence self-efficacy	The extent to which the smoker wants to smoke when being in a negative or emotional situation			
Habitual smoking abstinence self-efficacy	The extent to which the smoker wants to smoke when in a habitual or addictive situation			
Positive coping style	Whether smokers respond positively to life events	1=definitely yes 2=comparatively yes 3=neutral 4=comparatively no 5=definitely no	Trait coping style	0.90^20^
Negative coping style	Whether smokers respond negatively to life events			
Willingness to quit smoking scale score	The extent to which smokers want to quit	Continuous variables	Willingness to quit smoking	0.919^13^
Cessation effects indicators	7-day point prevalence smoking abstinence rate, at the time the smoker was surveyed, was used as the main reference indicator to determine whether they quit smoking	0=not quit 1=quit	Smoking cessation effects	
Change in smoking amount at 6 months	Change in smoking amount between the survey and the baseline survey at month 6 of the smokers’ enrollment	-1=increase in smoking amount 0=no change in smoking amount 1=reduction of 1–10 cigarettes 2=reduction of 11–20 cigarettes 3=reduction of 21–30 cigarettes 4=reduction of ≥31 cigarettes		

Manifest variable or observed variable is a variable that can be directly observed. Latent variable is usually a variable that cannot be directly observed and needs to be estimated with the help of an exogenous measure^21^.

*Trait Coping Style Questionnaire (TCSQ)*

The scale contains a total of 20 items on a 5-point Likert scale, with two factors, negative coping (NC) and positive coping (PC), and Cronbach’s α of 0.90, 0.89, and 0.78 for the full scale and the two sub-scales, respectively, indicating good internal consistency. The scale measures trait attributes that individuals have, and such attributes refer to health-related coping styles^20^.

*Self-administered Willingness to Quit Smoking Scale*

The scale was designed and developed by Xingming Li’s group to measure the willingness to quit smoking among smokers, which contains 10 items and is scored on a 5-point Likert scale, with a Cronbach’s α of 0.919, indicating good internal consistency. The results of the principal component analysis showed a total of one factor, and the confirmatory factor analysis showed that the factor’s structural validity is reasonable^13^. The scale measures the degree of an individual’s willingness to quit smoking. The higher the score, the stronger the willingness to quit.

*Cessation Effects Indicators*

Smoking cessation effects, an outcome indicator used to determine whether a smoker has quit smoking, was measured by 7-day point prevalence abstinence^24^ at the time the smoker was surveyed as the primary reference indicator to determine whether the smoker has quit smoking; 6-month change in smoking amount^25^, an outcome indicator reflecting the change in the smoker’s smoking amount after 6 months of enrollment, was measured based on the difference between the change in smoking at the baseline survey and the smoker’s smoking amount at the 6th month of enrollment. The assignment of each variable is shown in Table 1.

### Statistical analysis

Software EpidataV3.1 was applied to organize and summarize the questionnaire data and establish and manage the database, and the data were imported into software SPSS19.0 for processing and analysis. The chi-squared test was used to analyze the basic demographic characteristics of smokers and tobacco control interventions to ensure the comparability of individual psychological characteristics between quitters and non-quitters; the qualitative variables obtained from the questionnaire were described using the constituent ratio, the chi-squared test was used to compare the questions in the scales between groups, and non-parametric tests (Mann-Whitney U test) were used for abnormal distribution variables: positive smoking abstinence self-efficacy, negative smoking abstinence self-efficacy, habitual smoking abstinence self-efficacy, total smoking abstinence self-efficacy score, positive coping score, and negative coping score, were examined. The t-test was used to compare between groups on the total score of the normal distribution variables, willingness to quit, to examine its effect on whether smokers quit. Using 7-day point prevalence smoking abstinence rate measured at six months of follow-up as the outcome indicator. Variables that were statistically different in t-tests, chi-squared analyses, and non-parametric tests and had no covariance problems were: negative smoking abstinence self-efficacy score, habitual smoking abstinence self-efficacy score, and total self-efficacy score, were included in the dichotomous logistic regression analysis to explore the factors influencing smoking cessation effects among quitters.

The study hypothesized that smoking cessation self-efficacy, trait coping style and willingness to quit were all related to smoking cessation outcomes and would affect cessation outcomes (‘7-day point prevalence’ and ‘smoking amount after 6 months of enrollment’). Software Mplus 8.3 was used to construct structural equation models and conduct validation factor analysis based on the study context and survey data, with parameter estimation using the modified weighted least squares means and variance (WLSMV) methods, latent variable definition using the fixed-loading methods, and analysis using two-tailed tests^21^. In the confirmatory factor analysis of the structural equation model, in order to improve model identification, research participants with missing psychological trait variables were excluded from 413 study subjects, and 284 research participants were finally included, as shown in Figure 1. A value of α=0.05 was used as the test level for all statistical analyses, and differences were considered statistically significant at p<0.05. All tests were performed using a two-tailed test.

### Quality control

The intervention follow-up was conducted with the following protocols to ensure data quality: 1) One-to-one question-and-answer data survey by trained investigators using a uniformly designed questionnaire, with reviewers double checking the questionnaire; 2) Double recording of the questionnaire using software Epidata, proofreading inconsistent data one by one, and eliminating questionnaires with poor quality; 3) The inclusion and exclusion criteria of the study subjects were strictly reviewed, and the study subjects were required to register by real name to ensure the authenticity of the information. Informed consent was obtained from the study subjects and patient privacy was strictly protected; and 4) Carbon monoxide test was used to show the parts per million (ppm) carbon monoxide in the subjects’ exhaled breath to verify the study subjects’ tobacco use or not.

## RESULTS

### Basic demographic characteristics and tobacco control interventions

A total of 413 subjects were included in this study after screening valid questionnaires and data cleaning, including 375 (91.5%) male smokers; 119 (29.0%) were aged 50–59 years and 139 (33.8%) 60–69 years; 364 (88.6%) were married; the education level was mainly junior high and high school, with 221 (53.8%) in total; in terms of employment status, nearly half were retired, with 181 (44.5%) in total. The majority of the group had a monthly income 2001–6000 RMB (RMB: 1000 Chinese Renminbi about US$145, for 2018–2020), 118 (32.5%) 2001–4000 RMB, and 90 (24.8%) 4001–6000 RMB. There was no significant difference between those who quit smoking and those who did not quit smoking in terms of basic demographic characteristics (p>0.05), which indicates that there was no statistical association between success in quitting and gender, age, marital status, employment status, and monthly income. Table 2 presents the information of the subjects included in the study.

**Table 2:** Sociodemographic characteristics of community intervention subjects in Beijing, China, 2018–2020 (N=413)

*Characteristics*	*Successful smoking cessation group n (%)*	*Unsuccessful smoking cessation group n (%)*	*Total n*	*χ^2^*	*p*
**Gender**					
Male	116 (30.9)	259 (69.1)	375	0.559	0.455
Female	14 (36.8)	24 (63.2)	38		
**Age** (years)					
20–29	4 (28.6)	10 (71.4)	14		
30–39	12 (24.0)	38 (76.0)	50		
40–49	22 (35.5)	40 (64.5)	64	7.917	0.161
50–59	29 (24.4)	90 (75.6)	119		
60–69	52 (37.4)	87 (62.6)	139		
70–79	11 (40.7)	16 (59.3)	27		
**Marital status**					
Not married	3 (12.0)	22 (88.0)	25		
Married	121 (33.2)	243 (66.8)	364	5.605	0.231
Divorced	4 (26.7)	11 (73.3)	15		
Widowed	2 (28.6)	5 (71.4)	7		
**Education level**					
Primary school and lower	4 (18.2)	18 (81.8)	22		
Junior and senior high school	69 (31.2)	152 (68.8)	221	2.267	0.322
College and higher	57 (33.9)	111 (66.1)	168		
**Employment status**					
Unemployed	6 (42.9)	8 (57.1)	14		
Employed	56 (26.4)	156 (73.6)	212	5.903	0.052
Retired	67 (37.0)	114 (63.0)	181		
**Monthly income** (RMB)					
≤2000	18 (39.1)	28 (60.9)	46		
2001–4000	30 (25.4)	88 (74.6)	118		
4001–6000	32 (35.6)	58 (64.4)	90	4.255	0.513
6001–8000	12 (29.3)	29 (70.7)	41		
8001–10000	9 (32.1)	19 (67.9)	28		
>10000	14 (35.0)	26 (65.0)	40		
**Hukou household-registration**					
Urban	106 (31.0)	236 (69.0)	342	0.937	0.333
Rural	21 (37.5)	35 (62.5)			
**Team group**					
Control	46 (28.7)	114 (71.3)	160	0.901	0.343
Test	84 (33.2)	169 (66.8)	253		

RMB: 1000 Chinese Renminbi about US$145 (2018–2020).

### Univariate association analysis of psychological traits and smokers’ cessation effects

*Smoking abstinence self-efficacy*

A total of 375 (96.1%) of the study subjects were daily smokers. As for a strong desire to smoke, 285 respondents (69.8%) when they were in an anxious and depressed mood, 264 respondents (66.1%) when they were very angry, 268 respondents (66.8%) when they were upset or defeated, 263 respondents (65.3%) in the morning after waking up, 223 respondents (55.3%) when they felt the need to refresh themselves, and 204 respondents (50.4%) when they felt that they have not smoked for a while.

The differences in smoking cessation effects were statistically significant (p<0.05) in terms of whether smokers wanted to smoke in the following situations: being very angry, waking up in the morning, feeling the need for refreshment, and feeling that they have not smoked for a while, with chi-squared values 10.602, 21.887, 10.790, and 15.835, respectively. The main influencing factors were concentrated on two contextual dimensions: negative smoking abstinence self-efficacy and habitual smoking abstinence self-efficacy, and the results of the nonparametric test (Wilcoxon rank sum test) showed that the difference between these two contextual dimensions was statistically significant (p<0.05), with u values 14375.500 and 13365.500, respectively (Table 3).

**Table 3:** Factors analysis of three contextual dimensions on whether community-based smoking cessation intervention subjects quit smoking (N=413)

*Dimension*	*Rank mean*		*u*	*p*
	*Successful smoking cessation group*	*Unsuccessful smoking cessation group*		
Positive smoking abstinence self-efficacy score	208.42	193.98	15633.500	0.239
Negative smoking abstinence self-efficacy score	216.67	189.66	14375.500	0.027
Habitual smoking abstinence self-efficacy score	231.08	186.60	13365.500	<0.01
Total smoking abstinence self-efficacy score	222.87	181.80	12675.500	<0.01

*Trait coping style and willingness to quit smoking*

The results of the non-parametric test (Wilcoxon rank sum test) showed that the differences between the two factors of positive coping and negative coping were not statistically significant (p>0.05) in the results of smoking cessation.

The lowest willingness to quit score was 10, and the highest score was 50, and the average willingness to quit score was 21.710 ± 8.052. The t-test results showed that the difference in the Willingness to Quit Smoking Scale score was statistically significant (p<0.05), with t-value of -2.578.

*Multi-factor association analysis of psychological traits and smoking cessation effects*

The 7-day point quit rate (cessation effect) measured at six months was used as the dependent variable, and the variables that were statistically different in the univariate analysis and did not have covariance issues were included in the binary logistic regression: self-efficacy score for smoking cessation in the negative/emotional scenario, self-efficacy score for smoking cessation in the habit/addiction scenario, and total intention to quit score.

The dichotomous logistic regression analysis of smokers’ cessation outcomes showed that the factors that influenced smokers’ cessation outcomes were in habitual smoking abstinence self-efficacy and willingness to quit smoking (p<0.05) (OR=0.77; 95% CI: 0.657–0.912; and OR=1.06; 95% CI: 1.008–1.118, respectively).

The results further showed that for every 1-level decrease in the habitual smoking abstinence self-efficacy score, the smoker had 0.68 odds more likely to quit; for every 1-level increase in the willingness to quit score, the smoker had 1.06 odds more likely to quit. Thus, the severity of the habit/addiction situation and the level of willingness to quit had a significant effect on cessation outcomes (Table 4). Model fit indicators are also shown in Table 5.

**Table 4:** Multi-factor analysis of the association between smoking cessation effects and their psychological traits among community-based smoking cessation intervention subjects in Beijing, China, 2018–2020

*Factors*	*β*	*S.E.*	*Walds*	*p*	*OR*	*95 % CI*
Habitual smoking abstinence self-efficacy score	-0.256	0.084	9.354	0.002	0.774	0.657–0.912
Total willingness to quit smoking score	0.060	0.026	5.119	0.024	1.061	1.008–1.118
Negative smoking abstinence self-efficacy score	0.017	0.078	0.048	0.827	1.017	0.873–1.185

Negative smoking abstinence self-efficacy score was used as an adjusted variable.

**Table 5:** Model fit indicators

*Fit index*	*Recommended values*	*Model indicators*	*Compliant*
ML χ^2^	The smaller the better	94.890	
df	The smaller the better	59	
χ^2^/df	1< χ^2^/df <3	1.794	Compliant
CFI	>0.9	0.975	Compliant
TLI	>0.9	0.968	Compliant
RMSEA	<0.08	0.046	Compliant
SRMR	<0.08	0.057	Compliant

RMSEA: root mean square of error of approximation. CFI: comparative fit index. TLI: Tucker-Lewis index. SRMR: standardized root mean square of residuals.

*Mechanism analysis of effect of psychological traits on smoking cessation effects among smokers*

**Figure 2:**
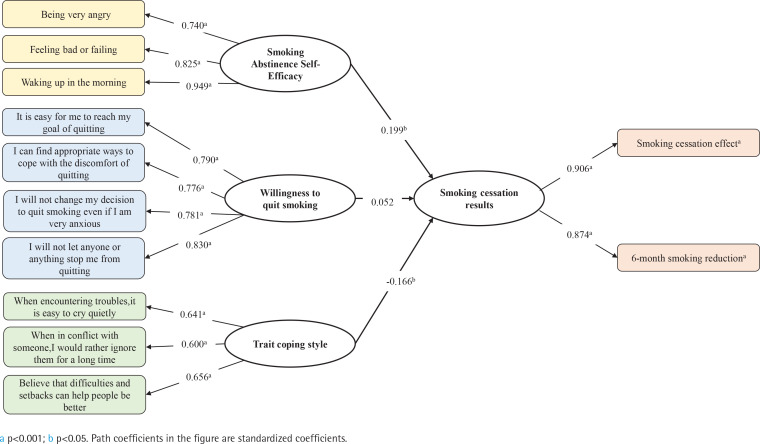
Paths of how psychological traits affecting on smoking cessation effects among smokers

Results of structural equation modeling of smoking cessation effects showed that: smoking abstinence self-efficacy was explained by ‘being very angry’ (β=0.740, p<0.001), ‘feeling bad or failing’ (β=0.825, p<0.001), and ‘waking up in the morning’ (β=0.949, p<0.001). Willingness to quit smoking was explained by ‘It is easy for me to quit’ (β=0.790, p<0.001), ‘I can find appropriate ways to cope with the discomfort of quitting’ (β=0.776, p<0.001), ‘I will not change my decision to quit smoking even if I am very anxious’ (β=0.781, p<0.001), and ‘I will not let anyone or anything stop me from quitting’ (β=0.830, p<0.001). Trait coping style was explained by ‘Prone to cry quietly in case of troubles’ (β=0.641, p<0.001), ‘Ignoring each other for a long time when in conflict’ (β=0.600, p<0.001), and ‘Believe that difficulties and setbacks can help people be better’ (β=0.656, p<0.001). The effect of smoking cessation was explained by ‘smoking cessation effect’ (β=0.906, p<0.001) and ‘6-month smoking amount reduction’ (β=0.874, p<0.001). ‘Smoking abstinence self-efficacy’ (β=0.199, p=0.002) and ‘trait coping style’ (β= -0.166, p=0.042) had an effect on smokers’ quitting, which further indicated that smoking abstinence self-efficacy had a significant influence on smoker’ cessation effect, i.e. the stronger the individual’s ability to refuse smoking when very angry, upset or feeling defeated, the less prone to smoking after waking up in the morning, and the more the smoker’s quit rate is improved. As for trait coping style, the less the negative coping style of wanting to cry when in trouble or ignoring each other for a long time when in conflict, the more the smoker’s quit rate is improved (Figure 2).

Further details of the study can be found in the Supplementary file.

## DISCUSSION

The World Health Organization (WHO) defines tobacco dependence as a substance-dependent mental disease which is directly related to a smoker’s psychological characteristics, and studies have shown that without external intervention only 3% of smokers are able to quit successfully due to their own perseverance. Most smokers can only be free from tobacco dependence through pharmacotherapy combined with psychotherapy, social support, and behavioral interventions^1,5,26^. We used survey data from adult smokers participating in community-based smoking cessation intervention projects in Beijing to describe the current psychological traits of smokers, analyze the differences in quit rates among people with different psychological traits, and investigate the relationship between influencing factors and the smoking cessation effects among smokers. The results showed that negative smoking abstinence self-efficacy, habitual smoking abstinence self-efficacy, negative coping style, and willingness to quit smoking had significant effects on their cessation outcomes.

### How smoking abstinence self-efficacy affects smoking cessation effects

Smoking abstinence self-efficacy is one of the factors that influence the smoking cessation effects. The results of binary logistic regression showed that smoking abstinence self-efficacy in habit/addiction situations had a negative effect on cessation, which is contradictory to the findings of existing studies that all contextual dimensions have a positive effect on cessation, for reasons to be further analyzed. The higher the individual’s smoking abstinence self-efficacy score, the higher the smoker’s confidence that he or she can curb the desire to smoke, and the better the cessation effects. Li^27^ found that high smoking abstinence self-efficacy can help quitters resist external influences and reduce smoking behavior, which in turn promotes smoking cessation. An increase in smoking abstinence self-efficacy can promote cessation and reduce the likelihood of relapse^28,29^. It is suggested that we should develop appropriate psychological interventions according to different dimensions; smoking behavior in the negative/emotional context shows that smokers use it as a way to regulate their emotions and stress, and smokers need to improve their ability to deal with negative emotions and learn the correct way to deal with emotions; and smoking behavior in the habit/addiction context is a habitual behavior, and they need to be helped to find alternative behaviors, and in severe cases, medication should be adopted.

### How trait coping style affects smoking cessation effects

The results of this study are consistent with the findings of Li^27^. The structural equation model was used to treat the difference in the smoking cessation effects among people with different coping styles when facing difficulties or unpleasantness. The reason for this difference is presumed to be the following: only two factors, positive coping and negative coping, were included in the logistic regression analysis used in this study, and too few dimensions led to insignificant differences in their effects on smoking cessation. In contrast, the structural equation model included all variables from the scales in the study, so the difference in smoking cessation effect was significant. Some studies have shown that psychological stress and self-tolerance are risk factors for smoking behavior. Negative coping is a sign of poor psychological tolerance, and this population is prone to accept addictive behaviors to reduce their negative emotions such as anxiety and dissatisfaction when facing stress^30^. However, it is clear that smoking is not a solution to the problem, and Parrott et al.^31^ found that for smokers choosing to smoke negatively to gain temporary relief can instead increase their psychological stress which can become more severe as nicotine dependence increases, leading to a vicious cycle.

### How willingness to quit smoking affects smoking cessation effects

The willingness to quit smoking is a key factor to improve the smoking cessation effect of smokers. Xu et al.^32^ found that probably because the quitters were in important stages such as starting a family or preparing for pregnancy, a higher percentage of quitters aged 18–40 years had a quit plan. Wang et al.^33^ noted that the percentage of voluntary quitters who had previous quit attempts had a quit plan had 8.986 odds higher than the percentage of voluntary quitters who had not tried to quit previously. Chen et al.^34^ found that the results of multi-factor logistic regression analysis revealed that the smoking cessation effect was poorer among those who had not tried to quit and those who quit after 30 days of preparation. Also, in the smoking cessation clinics in many places such as Shenzhen, Gansu and Beijing, it was found that the main reason for unsuccessful cessation was the smokers’ lack of willingness to quit^35-37^. In addition, smokers who were aware of the dangers of smoking, had strict anti-smoking rules in their units and had received advice from healthcare professionals to quit within a year of consultation, had a higher intention to quit smoking themselves^38^.

### Strengths and limitations

This study analyzed the effect of psychological traits on smoking cessation by using structural equation modelling based on a well-designed community intervention, which analyzed more comprehensively the mechanism between psychological factors and smoking cessation behavior from the perspective of biopsychosocial medicine model, which can be used to carry out a psychological assessment before smoking cessation intervention, so as to provide relevant reference for more accurate positioning of the target population. However, there are some limitations in the study, such as the lack of a corresponding psychological intervention program to implement in practice; the intervention program only started from the demand perspective, but did not investigate from the perspective of community workers, community health agency workers and smoking cessation volunteers, and lacked analysis of the supply perspective. The study did not exhaustively include all factors that may affect the outcome of smoking cessation and did not consider whether there is an indirect effect between factors.

## CONCLUSIONS

In this study, psychological traits were categorized into smoking abstinence self-efficacy, trait coping style, and willingness to quit, and the results showed that individual willingness to quit had a positive effect on smokers’ cessation outcomes, while smoking abstinence self-efficacy in habit/addiction situations had a negative effect. This suggests that smoking abstinence self-efficacy and willingness to quit are key factors that affect cessation outcomes.

## Data Availability

The data supporting this research are available from the authors on reasonable request.
