# Supplementary material for: Mechanism of community quitters’ psychological traits on their smoking cessation effects: Based on a study of community intervention
Source: Tob Induc Dis. 2023 May 26;21:70. doi: 10.18332/tid/162000 (PMC10210092; doi:10.18332/tid/162000)
Supplement: Supplementary file 1 [file TID-21-70-s1.pdf]

The Supplementary Material

Table 1 Dimensions in Smoking Abstinence Self-efficacy Scale

Dimension	Items and Content
Positive Smoking Abstinence Self-Efficacy (SASEP)	◯, 1Partying with friends;
	◯, 2Drinking tea, resting and chatting;
	◯, 3With spouse, or good friends who are smokers;
Negative Smoking Abstinence Self-Efficacy (SASEN)	◯, 1Feeling relatively anxious and depressed;
	◯, 2Being very angry;
	◯, 3Feeling bad or failing；
Habitual Smoking Abstinence Self-Efficacy (SASEH)	◯, 1Waking up in the morning;
	◯, 2Feeling the need for refreshment;
	◯, 3Feeling that have not smoked for a while

Table2 Factor analysis of variables in the Smoking Abstinence Self-Efficacy Scale on whether to quit smoking among community-based smoking cessation intervention subjects [n (%), n=413]

Factor	Category	Successful smoking cessation group	Unsuccessful smoking cessation group	Total	χ^2	*P*
Being very angry						
	extremely want to smoke	33（26.4）	92（73.6）	125		
	very want to smoke	35（25.2）	104（74.8）	139		

	somewhat want to smoke	30（40.0）	45（60.0）	75	10.602	0.031
	not really want to smoke	16（42.1）	22（57.6）	38		
	not want to smoke at all	10（45.5）	12（54.5）	22		
Waking up in the morning						
	extremely want to smoke	27（22.5）	93（77.5）	120		
	very want to smoke	37（25.9）	106（74.1）	143		
	somewhat want to smoke	26（36.1）	46（63.9）	72	21.887	$P<0.01$
	not really want to smoke	21（52.5）	19（47.5）	40		
	not want to smoke at all	15（53.6）	13（46.4）	28		
Feeling the need for refreshment						
	extremely want to smoke	29（35.4）	53（64.6）	82		
	very want to smoke	36（25.5）	105（74.5）	141		
	somewhat want to smoke	32（29.4）	77（70.6）	109	10.790	0.029
	not really want to smoke	16（36.4）	28（63.6）	44		

	not want to smoke at all	15（55.6）	12（44.4）	27		
Feeling that have not smoke for a while						
	extremely want to smoke	23（28.4）	58（71.6）	81		
	very want to smoke	30（24.4）	93（75.6）	123		
	somewhat want to smoke	36（28.6）	90（71.4）	126	15.835	$P<$ 0.01
	not really want to smoke	24（48.0）	26（52.0）	50		
	not want to smoke at all	13（54.2）	11（45.8）	24		

Table 3 Factor analysis of variables in Trait Coping Style Scale on whether community-based smoking cessation intervention subjects quit smoking [n(%), n=413]

Factor	Category	Successful smoking cessation group	Unsuccessful smoking cessation group	Total	χ^2	*P*
Be able to forget the unpleasantness as soon as possible						
	definitely yes	6（18.8）	26（81.3）	32		
	comparatively yes	8（24.2）	25（75.8）	33		
	neutral	35（38.5）	56（61.5）	91	7.616	0.111
	comparatively no	18（24.3）	56（75.7）	74		
	definitely no	59（33.9）	115（66.1）	174		
Usually look on the bright side						
	definitely yes	7（38.9）	11（61.1）	18		
	comparatively yes	7（41.2）	10（58.8）	17		

	neutral	12（19.0）	51（81.0）	63	6.120	0.190
	comparatively no	32（31.1）	71（68.9）	103		
	definitely no	68（33.5）	135（66.5）	203		
Ability to turn negatives into positives more quickly						
	definitely yes	6（18.8）	26（81.3）	32		
	comparatively yes	9（28.1）	23（71.9）	32		
	neutral	24（27.3）	64（72.7）	88	7.537	0.110
	comparatively no	28（18.3）	71（71.7）	99		
	definitely no	60（38.7）	95（61.3）	155		
Prone to cry quietly in case of troubles						
	definitely yes	65（32.3）	136（67.7）	201		
	comparatively yes	24（36.4）	42（63.5）	66		
	neutral	18（29.5）	43（70.5）	61	1.917	0.751
	comparatively no	10（27.8）	26（72.2）	36		
	definitely no	10（25.0）	30（75.0）	40		
Bystanders could easily cheer you up						
	definitely yes	15（30.6）	34（69.4）	49		
	comparatively yes	13（31.7）	28（68.3）	41		
	neutral	30（28.3）	76（71.7）	106	2.595	0.628
	comparatively no	25（27.8）	65（72.2）	90		
	definitely no	43（36.8）	74（63.2）	117		
Believe that difficulties and setbacks could help people be better						
	definitely yes	8（34.8）	15（65.2）	23		
	comparatively yes	2（9，5）	19（90.5）	21		
	neutral	17（30.9）	38（69.1）	55	4.994	0.288
	comparatively no	31（32.6）	64（67.4）	95		
	definitely no	68（32.5）	141（67.5）	209		

Recalling the unpleasant things they encountered for a long time						
	definitely yes	36（31.0）	80（69.0）	116		
	comparatively yes	29（31.9）	62（68.1）	91		
	neutral	24（30.8）	54（69.2）	78	1.070	0.899
	comparatively no	13（26.0）	37（74.0）	50		
	definitely no	24（34.8）	45（65.2）	69		
Prefer to be alone when distressed						
	definitely yes	17（37.0）	29（63.0）	46		
	comparatively yes	8（26.7）	22（73.3）	30		
	neutral	20（29.9）	47（70.1）	67	1.466	0.833
	comparatively no	31(33.7)	61（66.3）	92		
	definitely no	33（29.5）	79（70.5）	112		

Table 4 Analysis of two factors influencing whether community-based smoking cessation intervention subjects quit smoking [n(%),n=413]

Different Dimension	Rank Mean		u	*P*
	Successful smoking cessation group	Unsuccessful smoking cessation group		
Positive coping score	172.69	170.24	12275.500	0.831
Negative coping score	171.94	169.84	12311.500	0.855

Table 5 Factors analysis of variables in Willingness to Quit Smoking Scale on whether community-based smoking cessation intervention subjects quit smoking [n (%), n=413]

	Successful smoking	Unsuccessful smoking	t	*P*

	cessation group	cessation group		
Willingness to quit smoking scale score	19.98±7.073	23.62±8.038	-2.578	0.011

The reference values of the model fitting parameters are shown in Table 5, and the structural equation model fitting indices are $X^2/df = 1.794$, CFI = 0.975, TFI = 0.968, RMSEA = 0.046, SRMR = 0.057, suggesting that the structural equation model of smoking cessation effect has a good fitting effect.

Table 6 Analysis of reliability, convergent validity and differential validity

Dim.	Items	Item Reliability	Composite Reliability	Convergence Validity	Discriminate Validity			
		STD.LOADING	CR	AVE	F1	F2	F3	F4
F1	3	0.737～0.953	0.879	0.710	**0.843**			
F2	4	0.777～0.828	0.873	0.632	0.066	**0.795**		
F3	3	0.609～0.652	0.667	0.400	0.139	-0.188	**0.632**	
F4	2	0.721～1.098	0.923	0.863	0.179	0.097	-0.148	**0.929**

Note: The diagonal bold texts are the AVE square root values, and the lower triangle is the Pearson correlation of dimensionality.

Table 7 Analysis of research model hypothesis

DV	IV	Estimate	S.E.	Est./S.E.	P-Value	R^2	Hypothesis
F4	F1	0.199	0.065	3.051	0.002	0.065	Support
	F2	0.052	0.07	0.747	0.455		Not Support
	F3	-0.166	0.082	-2.035	0.042		Support

© 2023 Huo X. et al.
